# Improving Medical Q&A Matching by Augmenting Dual-Channel Attention with Global Similarity

**DOI:** 10.1155/2022/8662227

**Published:** 2022-04-05

**Authors:** Shi Li, Yaohan Yao

**Affiliations:** Information and Computer Engineering College, Northeast Forestry University, Harbin, China

## Abstract

The emergence of online medical question-answer communities has helped to balance the supply of medical resources. However, the dramatic increase in the number of patients consulting online resources has resulted in a large number of repetitive medical questions, significantly reducing the efficiency of doctors in answering these questions. To improve the efficiency of online consultations, a large number of deep learning methods have been used for medical question-answer matching tasks. Medical question-answer matching involves identifying the best answer to a given question from a set of candidate answers. Previous studies have focused on representation-based and interaction-based question-answer pairs, with little attention paid to the effect of noise words on matching. Moreover, only local-level information was used for similarity modeling, ignoring the importance of global-level information. In this paper, we propose a dual-channel attention with global similarity (DCAG) framework to address the above issues in question-answer matching. The introduction of a self-attention mechanism assigns a different weight to each word in questions and answers, reducing the noise of “useless words” in sentences. After the text representations were obtained through the dual-channel attention model, a gating mechanism was introduced for global similarity modeling. The experimental results on the cMedQA v1.0 dataset show that our framework significantly outperformed existing state-of-the-art models, especially those using pretrained BERT models for word embedding, improving the top-1 accuracy to 75.6%.

## 1. Introduction 

The medical question-answer (QA) community facilitates convenient patient consultations and has accumulated large amounts of data. However, compared with the rapid increase in the number of questions, the doctors' scale is quite limited. Most online question-answer communities are not designed with maintainability in mind, although maintainability prediction techniques have improved considerably [1]. To significantly reduce doctors' workload, improve maintainability, and improve patient experience in the online medical QA community, it is necessary to design a method that automatically selects the best matching answer to a patient's question based on existing medical formal reply records.

**Figure 1:**
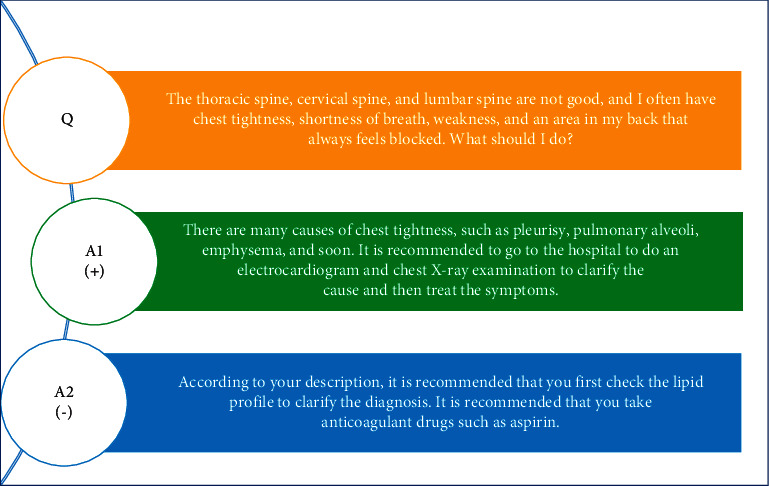
An example of a Chinese medical question-answer selection. Note: (+) indicates the ground truth answer and (−) indicates a negative answer.

As shown in Figure 1, unlike QA matching in open domains, medical QA contains more professional words; thus, it is difficult for general matching models to fully capture text features. In addition, patients' questions tend to be relatively brief, and the contextual information has no fixed pattern, which causes noise words to present new challenges in the overall text analysis. The purpose of this paper is to solve the problem of medical QA matching, selecting the best answer from the answer candidates. In recent years, deep QA matching models have been divided into two types: representation-based models and interaction-based models. Compared with representation-based models for question-answer matching, interaction-based models have been widely studied because they address the shortcomings of text representation, such as emphasizing the construction of the presentation layer and the loss of semantic focus. However, interaction-based models generally carry out interactive matching between words in the input layer. Although they have a better understanding of semantic focus, they ignore global similarity information such as syntax and intersentence contrast, as well as the effect of noise words on interactive matching. It is important to note that medical QA text is highly relevant to the profession, which amplifies the shortcomings of interaction-based models and poses a considerable challenge to feature extraction.

To solve the above problems, we constructed a dual-channel attention with global similarity (DCAG) framework based on interactive representations to improve medical QA matching. The framework consists of several components, including a bidirectional recurrent neural network, soft attention interactions, self-attention enhancement, and gating mechanisms. The bidirectional recurrent neural network learns context meaning from a sentence and extracts global sequence information, while the soft attention interaction generates similarity-weighted sentences from questions and answers and extracts interactive information. To reduce the influence of “noise words” in sentences and extract keywords, we introduced a self-attention enhancement to give different weights to each word in the question-answer sentences. Moreover, a gating mechanism combines the interactive sentence representations and keyword information, carrying out the final global similarity modeling to enrich the information and avoid performance degradation. We used the Chinese medical dataset cMedQA v1.0 to validate the proposed model. The relevant dataset was constructed with a positive example and several negative examples and was modeled as a ranking problem. Thus, this paper has two main contributions: (1) The dual-channel attention network fully captures interactive information between questions and answers as well as keyword information, extracts the complicated semantic relationship between the question-answer pairs, and reduces the impact of noise words. (2) The features obtained by the dual-channel attention network are combined with a gating mechanism to construct the global matching information.

The remainder of this paper is organized as follows.  Section 2 briefly summarizes related work.  Section 3 introduces our proposed DCAG framework in detail. The experimental results on our proposed framework and other competitive models are presented in Section 4. The effect of *k* values, the importance of new components in our model, and the limitations of the experiment are discussed in Section 5. Finally, the conclusions and future work are discussed in Section 6.

## 2. Related Work

Previous work on QA matching can be divided into two categories: traditional approaches and deep learning approaches. Traditional approaches have relied on information retrieval, manual rules, or shallow machine learning models. These models, which include TF-IDF [2] and BM25 [3], generally calculate the matching degree of a token at the word level. However, these models are incapable of handling cases in which there is a semantic correlation between the question and the answer, but the text does not match. In addition, in traditional approaches, feature extraction strategies and manual rules are not very flexible, resulting in poor generalization ability [4–7]. With the development of deep learning, researchers have proposed a series of nonlinear mappings combined with convolutional neural networks (CNNs), long short-term memory (LSTM) networks, and other structures that have yielded promising results in extracting the deep semantic feature representations of texts.  Table 1 summarizes the recently proposed text matching models. Below, we provide an overview of recent research on deep learning in QA matching.

### 2.1. Representation-Based Models for Question Answering

The first representation-based model adopted the Siamese networks proposed by Yann et al. [19]. The questions and answers were represented by low-dimensional vectors through a series of deep neural networks, and then the similarity between the questions and answers was calculated. Hu et al. [20] proposed two network architectures that use convolution models to learn sentence representations, which was the first work to use neural networks to solve general sentence matching problems. Qiu et al. proposed a vector representation model based on multilayer CNNs to generate questions and answers that uses *k*-max pooling and CNNs to train long sentences, which was especially suitable for longer answers and was applied in the English open-domain QA community [8]. Later, to explore the impact of different CNN structures on accuracy, Feng et al. [9] designed different CNN structures by altering the hidden layer and the convolutional layer, as well as the number of convolution kernels. Due to the insufficient content embedding capabilities of CNNs and max pooling, Tan et al. [21] utilized recurrent neural networks (RNNs) and their variant, long short-term memory networks [10], to better capture sequence information and learn sentence-level representations.

Although the representation-based models described above fully utilize the advantages of deep neural networks to extract semantic information, all the above studies were related to English texts and applied to the open-domain QA community. When these methods are used to directly process Chinese-specific questions and answers, their proposed method may significantly reduce performance because the specific domain QA text (especially in the Chinese medical field) is highly related to the profession.

For studies on medical QA matching, Zhang et al. [11] introduced character-level embedding to avoid Chinese word segmentation in medical texts and proposed multiscale CNNs to extract contextual information at different levels of granularity. To validate their framework, they constructed cMedQA v1.0, a new Chinese community medical text corpus. Ye et al. [12] proposed a multilayer composite convolutional neural network that went beyond stacking multiple convolutional neural networks. Instead, they extracted semantic information from each level to enrich the final vector representation. Excellent performance has been achieved on the cMedQA v1.0 dataset.

To simulate the abundant relationships between questions and answers, Zhang et al. [13] proposed different neural network architectures based on the advantages of CNNs and gated recurrent units (GRUs), including the BiGRU-CNN hybrid model, which significantly outperformed state-of-the-art methods on the cMedQA v1.0 dataset.

However, in terms of extracting features from sentences, the above representation-based model does not effectively use interaction information between texts and ignores feature matching between questions and answers, resulting in limited performance improvement.

### 2.2. Interaction-Based Models for Question Answering

In addition to considering the input representation, various interactive matching features between questions and answers can also be used to match as much granular information as possible. To obtain the interactive information between sentences, attention mechanisms are generally used to construct interaction matching matrices, which have been proven to be effective in natural language inference, response selection, textual entailment tasks, and sentence semantic matching [14, 15, 22, 23].

Tan et al. [16] proposed an attentive LSTM model to verify the effectiveness of the attention mechanism in answer selection tasks. The model dynamically adjusts the answer to a question by multiplying it by the softmax weight of the question. To avoid a lengthy answer with words unrelated to the current question, the more important words in the answer are given more weight.

Yin et al. [17] also used attention mechanisms in their early attempts at matching tasks. Attention was introduced in the convolutional and pooling layers, and the interaction between sentences was integrated into the CNN. Their method proved that interdependent sentence pair representations are more powerful than isolated sentence representations.

Because attention was added after the RNN in the previous study, words in later time steps were more likely to receive larger weights. As a result, Wang et al. [24] proposed three IARNN structures, with attention added before or within the RNN to obtain important information while avoiding weight imbalances. Chen et al. [22] and Wang et al. [25] calculated the interactive information between two sentences and then aggregated and matched the information units obtained at different time steps. The matching results were then transformed into a vector with a neural network (CNN or LSTM) and matched. This method captured finer granularity interactive features between two sentences.

In medical question-answer retrieval models, questions are generally short, and answers are composed of multiple sentences, indicating that the answer has an internal substructure. Therefore, Zhu et al. [26] calculated the attention of the answer within and between sentences, and the obtained vector representation was used to determine the similarity with the question.

However, the approaches described above generally use the single-attention model to obtain two-sentence interaction information and keyword information, which performs well on English datasets and Chinese datasets in general domains. When they are directly transferred to the medical domain, they are likely to suffer significant performance degradation.

Inspired by previous deep learning studies in general and specific fields (such as the medical field), we propose a dual-attention with global similarity framework for Chinese medical QA that combines character embedding, bidirectional recurrent neural networks, soft attention interactions, self-attention enhancement, and gating mechanisms.

### 2.3. Proposed Method

**Figure 2:**
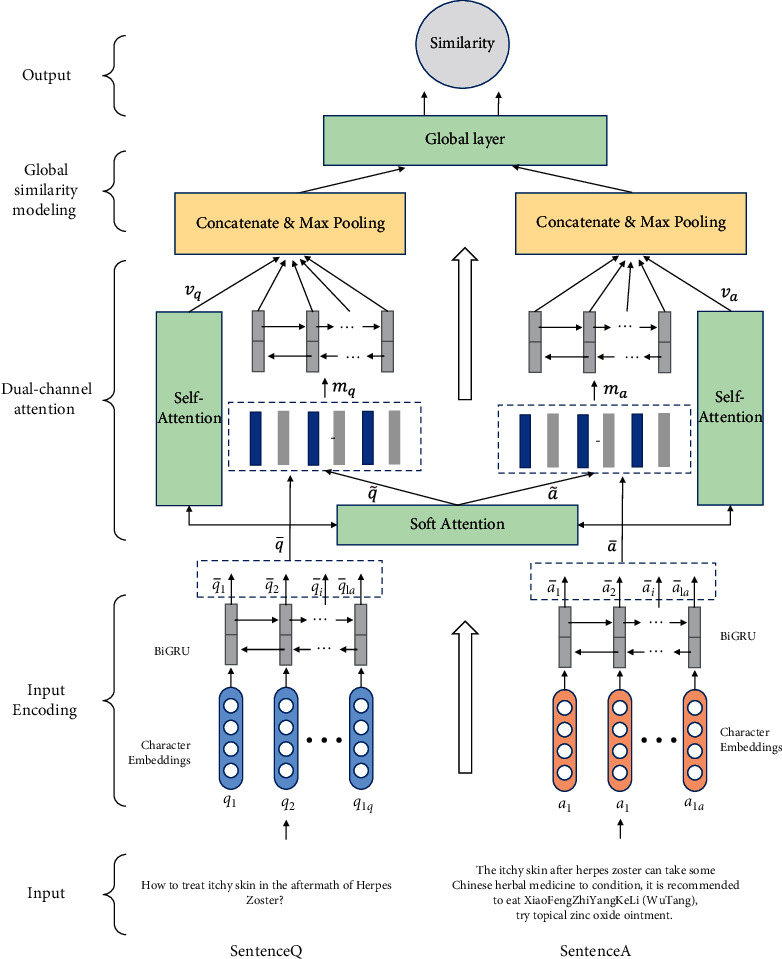
The architecture of the dual-channel attention with global similarity for medical QA matching.

Figure 2 illustrates our dual-attention framework with global similarity. In this study, we designed a dual-channel attention framework to capture the complex relationship between QA pairs. To express the comprehensive semantic relationships and characteristics of the QA pairs, we introduced a gate mechanism to model the global similarity. This section describes the details of the proposed DCAG framework. First, question and answer represented by character embedding are used as the structural input of the model, with the character embedding encoded by the pretrained model BERT or GloVe. Then, to better understand the sentence-level representation, the RNN-GRU variant is applied. After we obtain the semantics of the question and answer, we use the soft attention and self-attention layers to extract the interactive features between the question and the answer, as well as the key information features of the question and answer. Finally, the dual-channel attention output is spliced, and a gating mechanism is used to model the global similarity to improve the performance of the system.

#### 2.3.1. Input Encoding Layer

In contrast to English text processing, Chinese word segmentation is required prior to applying word embedding. Poor word segmentation has a serious impact on the overall performance of the pipeline, especially in fields such as medicine. Zhang et al. [11, 27] proved that, for the professional terms found in medical texts, the effect of character embedding was significantly better than that of word embedding. Thus, we implemented character embedding to reduce the adverse effects of word segmentation errors.

Consider a question sentence *S*_*q*_=[*s*_1_, *s*_2_,…*s*_*l*_*q*__] and a candidate answer *S*_*a*_=[*s*_1_, *s*_2_,…*s*_*l*_*a*__], where *l*_*q*_ and *l*_*a*_ are the number of characters in the question and answer, respectively, and *s*_*i*_ denotes the key value of each character-level vector in the vocabulary. We used pretrained word vectors, that is, character-level vectors trained on the relevant corpus in advance with GloVe [28] or BERT [29], to obtain fixed embedding for each character. After the embedding layer, *S*_*q*_ and *S*_*a*_ were represented as *E*_*q*_=[*q*_1_, *q*_2_,…*q*_*l*_*q*__] and *E*_*a*_=[*a*_1_, *a*_2_,…*a*_*l*_*a*__], where *E*_*q*_ ∈ *R*^*lq×dc*^ and *E*_*a*_ ∈ *R*^*la×dc*^ are character embedding for the question and answer, and the dimension of each character embedding *q*_*i*_ or *a*_*i*_ was *d*_*c*_. To obtain the context information of the whole character sequence in both directions, we used BiGRU to encode the character, and its output vector contained bidirectional sequence characteristics. We first used this vector to encode inputs *E*_*q*_ and *E*_*a*_ (equations (1) and (2)). BiGRU learned how to represent both a word (e.g., *q*_*i*_) and its context. To save the notations for later use, we wrote ${\bar{q}}_{i}$, the hidden (output) state generated by the BiGRU at time *i*, over the input sequence *E*_*q*_. The same was applied to ${\bar{a}}_{j}$:

(1) $$\begin{array}{c} {\bar{q}}_{i}=\text{BiGRU}\left(E_{q},i,\right),\;\forall i\in \left[1,\ldots ,l_{q}\right], \end{array}$$

(2) $$\begin{array}{c} {\bar{a}}_{j}=\text{BiGRU}\left(E_{a},j,\right),\;\forall j\in \left[1,\ldots ,l_{a}\right]. \end{array}$$

In the model, because the GRU has fewer parameters and converges more easily, its performance is comparable to that of the LSTM network. As a result, we choose to use the GRU [30] instead of the LSTM [10].

#### 2.3.2. Dual-Channel Attention Layer

**Figure 3:**
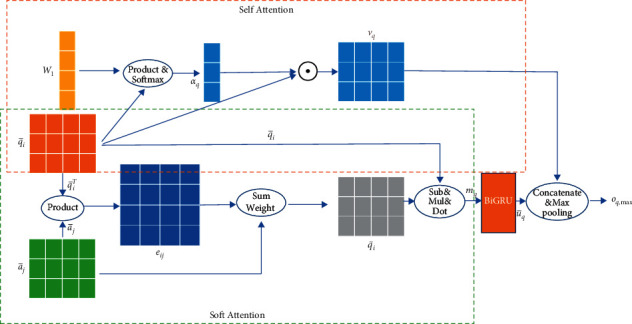
The whole process of the dual-attention layer for question *q* under the *i*-th single perspective.

We used soft attention and self-attention to capture the complex semantic information between the questions and answers of the output of the bidirectional sequence encoding, as well as the keyword information.  Figure 3 illustrates the dual-attention layer process for question *q* under the *i*-th single perspective. The soft alignment channel computed the attention weights as the similarity of a hidden state tuple 〈*q*_*i*_, *a*_*j*_〉 between the questions and answers with equation (3). In general, the character vectors of the questions and answers were multiplied to obtain the weight of the connection between each character.

(3) $$\begin{array}{c} e_{ij}={\bar{q}}_{i}{}^{T}{\bar{a}}_{j}. \end{array}$$

The character-level interactive feature vector representations between the questions and answers were determined by the attention weight *e*_*ij*_ computed above, as well as the bidirectional sequential encoding character hidden state of the question-answer pair, that is, ${\bar{q}}_{i}$, ${\bar{a}}_{j}$ (which encoded the word itself and its context), with the following equation:

(4) $$\begin{array}{c} {\tilde{q}}_{i}=\sum_{j=1}^{l_{q}}\frac{\exp\left(e_{ij}\right)}{\sum_{k=1}^{l_{q}}\exp\left(e_{ik}\right)}{\bar{a}}_{j},\;\forall i\in \left[1,\ldots ,l_{q}\right], \end{array}$$

(5) $$\begin{array}{c} {\tilde{a}}_{j}=\sum_{i=1}^{l_{a}}\frac{\exp\left(e_{ij}\right)}{\sum_{k=1}^{l_{a}}\exp\left(e_{kj}\right)}{\bar{q}}_{i},\;\forall j\in \left[1,\ldots ,l_{a}\right], \end{array}$$

where ${\tilde{q}}_{i}$ is a weighted summation of ${\left\{{\tilde{a}}_{j}\right\}}_{j=1}^{l_{a}}$. Intuitively, each character vector in the answer can be used to represent each character in the question based on the weight, resulting in the sequential calculation of a new interactive feature representation sequence. The same was performed for each word in the answer with equation (5).

In the soft attention channel, to improve the collected interactive feature information, we computed the difference and the elementwise product for both $\langle \bar{q},\tilde{q}\rangle$ and $\langle \bar{a},\tilde{a}\rangle$. First, the difference and similarity information between the output of the original bidirectional sequential encoding and the output after the soft attention interaction was obtained with this operation. Then, this information was concatenated with the original vectors, $\bar{q}$ and $\tilde{q}$ or $\bar{a}$ and $\tilde{a}$, respectively. The enhanced interactive information between the question and the answer was expressed as follows:

(6) $$\begin{array}{c} m_{q}=\left[\bar{q};\tilde{q};\bar{q}-\tilde{q};\bar{q}⊙\tilde{q}\right], \end{array}$$

(7) $$\begin{array}{c} m_{a}=\left[\bar{a};\tilde{a};\bar{a}-\tilde{a};\bar{a}⊙\tilde{a}\right]. \end{array}$$

Since the vector connection operation multiplied by the number of parameters, to prevent overfitting due to too many parameters, *m*_*q*_ and *m*_*a*_ were passed through a linear mapping layer, and the dimension was projected from 4*∗*2*∗* hidden size to the hidden size. Then, the BiGRU was used to extract the context features of the enhanced interaction information:

(8) $$\begin{array}{c} u_{q,i}=\text{BiGRU}\left(m_{q},i\right),\;\forall i\in \left[1,\ldots ,l_{q}\right], \end{array}$$

(9) $$\begin{array}{c} u_{a,j}=\text{BiGRU}\left(m_{a},j\right),\;\forall j\in \left[1,\ldots ,l_{a}\right]. \end{array}$$

While the soft attention calculation obtained the enhanced interactive information representation, the outputs $\bar{q}$ and $\bar{a}$ of the bidirectional sequential encoding were sent to the self-attention channel. First, we calculated the weight of each character in the question vector $\bar{q}$ and the answer vector $\bar{a}$. The specific calculation formulas were as follows:

(10) $$\begin{array}{c} k_{q}=\bar{q}W1,\\ k_{a}=\bar{a}W2,\\ \alpha_{q}=\mathrm{softmax}\left(k_{q}\right),\\ \alpha_{a}=\mathrm{softmax}\left(k_{a}\right), \end{array}$$

where $\bar{q}\in R^{l_{q}\times 2d_{c}}$ and *W*_1_ ∈ *R*^2*d*_*c*_×1^ represent the parameter matrices of the question; $\bar{a}\in R^{l_{a}\times 2d_{c}}$ and *W*_2_ ∈ *R*^2*d*_*c*_×1^ represent the parameter matrices of the answer; softmax(·) indicates the nonlinear activation function; and *α*_*q*_ ∈ *R*^*l*_*q*_×1^ and *α*_*a*_ ∈ *R*^*l*_*a*_×1^ represent the weight distributions of each character in the question-answer sentences, respectively. Then, the weights were multiplied by the question vector $\bar{q}$ and the answer vector $\bar{a}$ (equations (11) and (12)) to generate a new question vector *v*_*q*_ and answer vector *v*_*a*_ that enhance the keyword information.

(11) $$\begin{array}{c} v_{q}=\alpha_{q}⊙\bar{q}, \end{array}$$

(12) $$\begin{array}{c} v_{a}=\alpha_{a}⊙\bar{a}. \end{array}$$

#### 2.3.3. Global Similarity Modeling Layer

Before the global similarity modeling layer, we concatenated the enhanced interactive information vectors *u*_*q*_ and *u*_*a*_ and the enhanced keyword information vectors *v*_*q*_ and *v*_*a*_ to compute the max pooling:

(13) $$\begin{array}{c} o_{q,\max}=\overset{l_{q}}{\underset{i=1}{\max}}\left[u_{q,i};v_{q,i}\right], \end{array}$$

(14) $$\begin{array}{c} o_{a,\max}=\overset{l_{a}}{\underset{j=1}{\max}}\left[u_{a,j};v_{a,j}\right]. \end{array}$$

After max pooling, the enhanced question-answer information was output to the global similarity modeling layer, and a gating mechanism was introduced to perform global similarity modeling. When designing the global similarity modeling layer, we used the SLQA model proposed by DAMO Academy in the field of machine reading, connected the question and answer representations in series, and performed nonlinear transformations (equation (15)). The primary purpose was to improve the integration of the interactive information representations of the questions and answers.

(15) $$\begin{array}{c} m\left(Q,A\right)=\tanh\left(W_{f}\left[Q:A;Q\circ A;Q-A\right]+b_{f}\right). \end{array}$$

The vector *m* in the fusion representation was combined with the original context representation (enhanced question-answer information) with a gating mechanism. The fusion result of the final question and answer is expressed as follows:

(16) $$\begin{array}{c} Q^{\prime }=g\left(o_{q,\max},o_{a,\max}\right).m\left(o_{q,\max},o_{a,\max}\right)+\left(1-g\left(o_{q,\max},o_{a,\max}\right)\right).o_{q,\max},\\ A^{\prime }=g\left(o_{a,\max},o_{q,\max}\right).m\left(o_{a,\max},o_{q,\max}\right)+\left(1-g\left(o_{a,\max},o_{q,\max}\right)\right).o_{a,\max}, \end{array}$$

where *g* is a gating function.

Finally, we used the cosine distance to calculate the similarity between the question and the answer as

(17) $$\begin{array}{c} \text{sim}\left(Q^{\prime },A^{\prime }\right)=\text{cosine}\left(Q^{\prime },A^{\prime }\right)=\frac{\left\|Q^{\prime }.A^{\prime }\right\|}{\left\|Q^{\prime }\right\|.\left\|A^{\prime }\right\|}, \end{array}$$

where ‖·‖ is the vector length.

#### 2.3.4. Objective Function

While training the deep neural networks, the pointwise learning method was used to solve the sorting problem, and the training samples were organized into multiple (*q*_*i*_, *a*_*i*_^+^, *a*_*i*_^−^) triplets, each of which contains a question *q*_*i*_, a correct answer *a*_*i*_^+^, and a random wrong answer *a*_*i*_^−^. The goal of our training was to calculate the similarity relationship between two samples by maximizing the score of the positive sample Sim(*q*_*i*_, *a*_*i*_^+^) and minimizing the score of the negative sample Sim(*q*_*i*_, *a*_*i*_^−^). Therefore, the max-margin loss function was used as the objective function to train the neural networks. The formula is as follows:

(18) $$\begin{array}{c} L=\max\left\{0,M-\text{sim }\left(q_{i},a_{i}^{+}\right)+\text{sim }\left(q_{i},a_{i}^{-}\right)\right\}, \end{array}$$

where the margin value *M* is a constant. During training, Sim(*q*_*i*_, *a*_*i*_^+^) − Sim(*q*_*i*_, *a*_*i*_^−^) > *M* indicates that the similarity of the correct answer is much larger than that of the wrong answer; thus, the cost is zero, and no updates are required.

## 3. Experiments and Results

To validate our method, we compared the proposed model with four competitive baselines on the cMedQA v1.0 dataset [11] (https://github.com/zhangsheng93/cMedQA). The model was implemented using PyTorch, and the source code and additional information are available on GitHub (https://github.com/1076325946/Medical-QA-selection).

### 3.1. Dataset

We evaluated the medical text matching power using the cMedQA v1.0 dataset, which was constructed by Zhang et al. [11]. Questions are submitted by users, which are then answered by professionally trained doctors. Each question is usually answered by several doctors. To the best of our knowledge, this is the first publicly available dataset based on Chinese medical questions and answers. As shown in Table 2, the dataset includes three parts: (1) the training set, (2) the development set, and (3) the test set. The training set is used to train the model parameters, the development set is used to select the hyperparameters of the model for fine tuning, and the test set is used to evaluate the generalization ability of the model. The dataset contains 54,000 questions and 101,743 answers. Our proposed model divided the questions and answers into Chinese characters, with an average of 119 characters in each question and 212 characters in each answer, with 4979 tokens in the total vocabulary.

### 3.2. Evaluation Metric

We validated the proposed models using top-k accuracy measures. The top-k accuracy (ACC@*k*) is frequently used in the field of information retrieval. The top-k accuracy (ACC@*k*) is defined as follows:

(19) $$\begin{array}{c} \text{ACC}@k=\frac{1}{N}\sum_{i=1}^{N}1\left[a_{i}\in C_{i}^{k}\right], \end{array}$$

where *a*_*i*_ is the ground truth answer for question *q*_*i*_ and *C*_*i*_^*k*^ is the candidate set with the top-k highest similar answers for question *q*_*i*_. The function 1[*·*] ⟶ is an indicator function, with a value of 1 when the condition in the square brackets is true and a value of 0 if the condition is false.

Unlike traditional information retrieval [31], for a medical question, it is necessary to return an answer that is as relevant as possible, rather than a ranked list of candidate answers. Therefore, we adopted top-1 accuracy (ACC@*k*) in this work as our performance measure, which is a computationally demanding measurement.

### 3.3. Baselines

To prove the superiority of our model, we compared our model with various baselines. The following state-of-the-art technologies were used as baselines to evaluate our model:

- SingleCNN: SingleCNN uses a fixed-size sliding window to capture local features and reduces the feature dimension of the convolutional layer output through a pooling operation. SingleCNN can be used to capture the unilateral characteristics of QA pairs. The capabilities and performance of the SingleCNN are limited.
- MultiCNN: MultiCNN framework can capture various sentence features. The input sentence is processed by different sized convolution kernels, and the maximum pool output is spliced to produce a more reasonable sentence representation. MultiCNNs are good at capturing a variety of low-level n-gram features at the same time and determining broader relationships.
- Multiscale attention interaction networks: this framework extracts information from each layer of a neural network and connects the obtained high-level dimensional semantic features, not only obtaining a semantic representation of character-level embedding but also enriching the semantic relationships between the questions and answers.
- BiGRU-CNN: this model was proposed by Zhang et al. [13] and achieved the best performance on the cMedQA v1.0 dataset. The BiGRU-CNN combines the feature extraction advantages of BiGRU and CNN. The BiGRU can capture order information and long-distance dependency in a sentence, while the CNN can capture position-invariance features.

### 3.4. Experimental Settings

For preprocessing, we used pretrained embedding to convert the tokens in the question-answer sentences into character-level vectors. The pretraining embedder GloVe uses Chinese character embedding from Chinese word vectors [32] and has a dimension of 300. The pretraining embedder BERT used the Chinese version of Google's BERT-Base model [29] and has a dimension of 768. We set the maximum sentence length to *l* = *l*_*q*_ = *l*_*a*_ = 400, which is the point at which we apply zero padding or truncation to all questions and answers.

In this study, the output shape of the BiGRU was 400 (hidden size = 200 for each direction). When training the neural networks, the margin value *M* of the loss function was set to 0.05, the dropout was set to 0.1, and an AdaGrad optimizer with a batch size of 64 and an initial learning rate of 0.001 was used. Because the gradient descent method is very sensitive to the selection of the initial position point when solving for the local optimum, the random seed was set to 1234 to ensure that each experiment was accurate.

## 4. Results

In this section, the experimental results of the proposed method are presented.  Table 3 summarizes the results for the different approaches on the cMedQA v1.0 dataset. The first column is the index, and the second shows the pretrained word embedding used in the experiment. The third column shows the models, which include the baseline models and our model. The fourth and fifth columns show the top-1 accuracies on the development and test sets, respectively. DCAG is our proposed dual-channel attention model with global similarity, and the remaining models are the baseline models, including the BiGRU-CNN model proposed by Zhang et al. [13], which achieved the best performance on the cMedQA v1.0 dataset. The proposed hybrid model, DCAG, achieved the best accuracy on the test dataset with pretrained BERT as the word embedder, demonstrating improvements of approximately 10.8%, 8.5%, and 7.1% over the MultiCNN, multiscale attentive interaction network, and BiGRU-CNN models, respectively. Our proposed DCAG model outperformed all baseline models, proving that it is reasonable and powerful to use a dual-channel attention method with global similarity to model the complex relationships and features between question-answer pairs.

Rows 1 to 5 of Table 3 show the results of the neural network model using pretrained GloVe word embedding. Compared to the models using pretrained BERT word embedding (Rows 6 to 10), the difference in the same model was between 2.4% and 3.4%. It is worth noting that our proposed model was 3.2% more effective on BERT than on GloVe, demonstrating the importance of keyword information extraction for highly specialized medical texts. Because GloVe has difficulty interpreting ambiguity and complexity in the sentences, a large amount of noise was generated during the input phase of the model. Although our model reduced noise and enhanced the key information, GloVe still performed worse than BERT on medical text. GloVe word embedding struggles to match BERT in terms of performance, but when training time and computational resources are taken into account, GloVe has an advantage.

Rows 6 to 7 show that the MultiCNN model had a better performance in the single-layer network than the SingleCNN model. This indicates that, compared with SingleCNN, MultiCNN uses convolutional kernels of different sizes to capture multiple n-gram features simultaneously, avoiding the limitations of single CNNs, which can only capture features on one side. However, the CNN approach only captures local location information and cannot extract contextual semantic information from character-level embedding.

Rows 8 to 9 show the results of the multilayer models based on the MultiCNN model (Row 7). The comparison results clearly show that the multiscale attentive interaction network and the BiGRU-CNN model performed better than the MultiCNN model. The multiscale attentive interaction network included multilevel composite attention interactions, which not only fully capture the semantic information from the character embedding but also extract the relevant relationships between questions and answers. This suggests that adding attention mechanisms is useful, as questions and answers themselves contain interrelated information. The BiGRU-CNN model fully combined the strengths of BiGRU and CNN in feature extraction, capturing sequential information and long-distance dependency as well as position-invariant features. Row 9 (BiGRU-CNN model) achieved the best performance among the two MultiCNN-based methods, demonstrating the advantages of including sequential semantic information.

When the DCAG model we proposed in Row 10 was compared with the above models, it was found that the performance of the DCAG model far exceeded those of the models in Rows 1 to 9. The comparison with the single-layer network (Rows 1–2 and 6–7) indicated that the multilayer network structure used in our DCAG model provided significant performance improvement for medical text matching. In contrast to the attention mechanism of the multiscale attentive interaction network (Rows 3 and 8), the DCAG model introduced dual-channel attention, which not only models the interaction information between questions and answers but also uses self-attention to weigh key information for both questions and answers. The results in Table 3 also demonstrate the effectiveness of dual-channel attention. It is worth noting that our DCAG model performed 7.1% better than the BiGRU-CNN model (Row 4), which had previously achieved the best performance on the cMedQA v1.0 dataset. Introducing dual-channel attention and replacing the multiscale CNN may have weakened the extraction of different local location features. However, this was not the case, which may be due to the introduction of the gating mechanism, which greatly reduced the loss of local information by modeling the gating of the global text vectors. The results of our analysis were also verified by the ablation experiments in the next section.

To summarize, our models performed better than single-layer and multilayer models. This shows that the new components of our model play an important role in extracting medical text, as thoroughly discussed in Section 5. Additionally, our model included not only BiGRU, which captures long-distance dependency relations, but also multilevel attention and gating mechanisms for global similarity. It performed better than previous models, proving that our dual-channel attention model with global similarity is useful for medical QA matching.

## 5. Discussion

In the previous section, we compared the proposed model with baseline models and used top-1 accuracy to assess the quality of the model. The discussion in this section was threefold to fully analyze the robustness, interpretability, and limitations of our model: (1) the effect of *k* values on the experimental effectiveness of top-*k* accuracy; (2) the impact of the new components of our model on the experimental accuracy; and (3) a discussion of the experimental results that do not match expectations.

### 5.1. Accuracy of Different *K* Values

**Figure 4:**
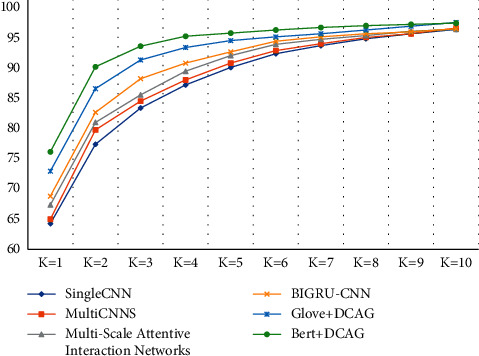
The effect of *k* values on the experimental effectiveness of the top-*k* accuracy.

As shown in Figure 4, our proposed DCAG framework outperformed the baseline model when the *k* value ranged from 1 to 10, regardless of whether pretrained BERT word embedding or pretrained GloVe word embedding was used, demonstrating the robustness of our model.

A *k* value of 3 indicates that a patient can accept the correct answer as one of the top 3 most similar candidates, at which point our model outperformed the BiGRU-CNN model. Interestingly, we found that when *k* was increased to 10, it was difficult to improve the accuracy of the model, and the difference between the use of GloVe and BERT in the model decreased. This shows that the difficulty in predicting the correct answer in the top 10 candidates was very low on the cMedQA v1.0 dataset, and it is clear that larger values of *k* are less reasonable and less desirable to the patient.

### 5.2. Ablation Analysis

To measure the importance of the new components in our model, we designed an ablation analysis experiment. We began with the original model, which was based on pretrained BERT embedding, and gradually removed these components to observe how the accuracy of the algorithm changed. Ablation analysis can help to identify the most important components.

Table 4 shows the performance of our model and its ablations. The first row is the result of our model, and the remaining rows are specific models that remained after ablation; the second column lists the key components of the model.

The experiment indicated that all components positively contributed to the performance of our model. The components sorted by their contribution in descending order are soft attention, self-attention, and the gating mechanism. Removing the soft attention interaction component alone has a more negative impact on the model performance than removing both the self-attention and gating mechanism components. This shows that questions and answers in medical QA pairs generally share a considerable amount of information. We should pay closer attention to the parts that contain interactive information between the questions and answers. As the components were removed, the top-1 accuracy of the remaining models on the test set gradually decreased, which indicates that each component in our model is positively correlated with the accuracy of the model. Another important result is that deleting multiple components seriously reduces the performance of the model, demonstrating that the multilayer neural network plays a crucial role in feature extraction.

The above ablation analysis shows that, in Chinese medical QA matching tasks, soft attention interaction, self-attention, and gating mechanisms are three key components that capture character-level semantic information.

### 5.3. Limitations of the Experiment

Although our method is capable of identifying high-quality answers to matching questions, there are still some limitations that need to be addressed.  Table 5 illustrates two errors observed during answer selection. For the questions in Table 5, our model selected incorrect answers. Both answers in the table refer to pregnancy, with the difference being that the correct answer contains the keyword information for the cesarean section. Specifically, the incorrect answer has some misspellings (剖腹产 is incorrectly written as 抛妇产). We infer that, due to the introduction of the dual-channel attention mechanism, the model overlearned more valid feature information, and incorrect spellings were overweighted, especially on smaller datasets. We also attempted to replace the dual-channel attention mechanism with a convolutional neural network. Although the number of error cases due to misspellings was reduced, the overall performance of the model was also considerably reduced. In terms of overall model performance, our proposed DCAG model still performed consistently on the data as a whole.

## 6. Conclusion

In this study, we proposed a dual-channel attention neural network framework with a gating mechanism for medical QA matching. The research in this paper focused on improvements based on the interaction representation framework, such as the addition of a dual-channel attention mechanism and the fusion of gating mechanisms for global similarity modeling, both of which aid in extracting more detailed information with lower complexity. To validate the robustness of the model, we explored the impact of the choice of k value on the model performance. In addition, we designed ablation experiments to test the components of the model to validate the interpretability from a different perspective. The final experimental results showed that our proposed DCAG model outperformed the baseline models in terms of learning sentence similarity on two different pretrained word vectors.

Our work has several limitations that can be addressed in future work. Although we captured global semantic information, we ignored global information such as syntax and intersentence comparisons. Particularly in medical texts, where sentences generally include descriptions of body parts and symptoms, fixed syntactic features and entity keyword extraction are likely to have a significant effect on text matching. In addition, the time cost and memory overhead were increased because of the introduction of the pretrained BERT model. Therefore, we present two directions for future work: (1) Specific syntactic features and semantic role markers should be added to the model. Syntactic features can be used to capture phrase structure and the hierarchical syntactic relationship between phrases. Semantic role markers can be used to identify the predicate and semantic subject in a question to determine the shallow semantic structure in the sentence. (2) Medical texts include professional terminology. This step involves enhancing the understanding of medical terminology. Specifically, a combination of character vectors and word vectors should be used. We intend to use Chinese electronic medical records as a corpus and BERT training vectors to generate word vectors.

## Figures and Tables

**Table 1:** Summary of recently proposed text matching models.

Model type	Ref	Datasets	Measures	Best neural network architecture
Representation-based model	9	Insurance QA	Accuracy	HL + CNN
	21	TREC-QA		CNN + GESD
	11	cMedQA	ACC@1	MultiCNNs
	12			Three-level composite CNNs
	13			BiGRU-CNN

Interaction-based model	22	SNLI	Accuracy	ESIM
	16	InsuranceQA	Accuracy	Attentive LSTMs
	17	TREC-QA		ABCNN
	24	WikiQA		IARNN
	25			BiMPM
	18	cMedQA	ACC@1	MCFN

**Table 2:** Statistics of the cMedQA v1.0 dataset.

	Ques	Ans	Ave. words per question	Ave. words per answer	Ave. characters per question	Ave. characters per answer
Train	50,000	94,134	97	169	120	212
Dev	2000	3774	94	172	117	216
Test	2000	3835	96	168	119	211
Total	54,000	101,743	96	169	119	212

**Table 3:** The top-1 accuracy results of the models.

Index	Pretrained embedding	Model	Dev (%)	Test (%)
1	GloVe	SingleCNN	64.5	64.1
2		MultiCNN	65.4	64.8
3		Multiscale attentive interaction networks	66.1	67.1
4		BiGRU-CNN	*68.5*	*68.5*
5		DCAG	**71.9**	**72.5**

6	BERT	SingleCNN	66.3	66.8
7		MultiCNN	67.5	67.2
8		Multiscale attentive interaction networks	70.1	70.5
9		BiGRU-CNN	72.4	71.9
10		DCAG	**75.1**	**75.6**

The best performance is boldfaced, and the state-of-the-art performance is italicized.

**Table 4:** Ablation analysis of our model.

	Components	Test (%)
1	BiGRU + soft attention + self-attention + gate	75.6
2	−Gate	−0.6
3	−Self-attention	−1.8
4	−Gate-self-attention	−3.4
5	−Soft attention	−6.3
6	−Gate-soft attention	−6.7
7	−Gate-soft attention-self-attention	−7.8

**Table 5:** Wrong answers in question-answer matching.

Question	How soon can I prepare for a pregnancy after a cesarean section?
Irrelevant answer	It is safer to consider pregnancy 3 months after stopping the medication, regardless of the external factors, for eugenic reasons. I hope my answer can help you.
Correct answer	Hello, it usually takes two years to recover from a cesarean section before you can get pregnant again.

## Data Availability

All data included in this study are available upon request to the corresponding author.
